# How mood tunes prediction: a neurophenomenological account of mood and its disturbance in major depression

**DOI:** 10.1093/nc/niaa003

**Published:** 2020-06-02

**Authors:** Julian Kiverstein, Mark Miller, Erik Rietveld

**Affiliations:** n1 Department of Psychiatry, Amsterdam University Medical Centre, Meibergdreef 9, 1105AZ Amsterdam South East, The Netherlands; n2 Department of Informatics, University of Sussex, Sussex House, Falmer, Brighton, BN1 9RH, UK; n3 Department of Philosophy/ILLC, University of Amsterdam, Oude Turfmarkt 141-3, 1012GC Amsterdam, The Netherlands; n4 Department of Philosophy, University of Twente, 7500AE Enschede, The Netherlands

**Keywords:** mood, predictive processing, depression, phenomenology, error dynamics

## Abstract

In this article, we propose a neurophenomenological account of what moods are, and how they work. We draw upon phenomenology to show how mood attunes a person to a space of significant possibilities. Mood structures a person’s lived experience by fixing the kinds of significance the world can have for them in a given situation. We employ Karl Friston’s free-energy principle to show how this phenomenological concept of mood can be smoothly integrated with cognitive neuroscience. We will argue that mood is a consequence of acting in the world with the aim of minimizing *expected* free energy—a measure of uncertainty about the future consequences of actions. Moods summarize how the organism is faring overall in its predictive engagements, tuning the organism’s expectations about how it is likely to fare in the future. Agents that act to minimize expected free energy will have a feeling of how well or badly they are doing at maintaining grip on the multiple possibilities that matter to them. They will have what we will call a ‘feeling of grip’ that structures the possibilities they are ready to engage with over long time-scales, just as moods do.

## Introduction

In what follows, we will propose a neurophenomenological theory of what moods are—a core, but often overlooked aspect of our first-person experience of the world. On the one hand, we put phenomenological ideas to work to show how mood structures lived experience. On the other hand, we set out to show how neurobiology can make sense of these phenomenological descriptions of mood in the terms of the free-energy principle (FEP) (Friston 2010). We call our account of mood, ‘neurophenomenological’ because it aims to smoothly integrate phenomenological description with cognitive neuroscientific explanation. Our use of the term ‘neurophenomenology’ (here abbreviated as ‘NP’) departs somewhat from that of Varela (1996). He originally introduced the term to describe an experimental method in which subjects in an experiment would be actively involved in identifying invariant and structural features of lived experience. In what follows, we do not draw our phenomenological descriptions of experience from experiments but instead base our descriptions on reflection on lived experience and its disturbance in psychopathology. In developing a neurophenomenological account of mood, we take as our starting point the phenomenological concept of an agent’s tending towards an optimal grip on the possibilities that matter to them (Merleau-Ponty 1962/2012; Dreyfus 2005; Rietveld 2008; Bruineberg & Rietveld 2014). In earlier work, we have shown how this phenomenological concept can be understood as a consequence of anticipatory biological processes on the basis of which living systems minimize their own free energy—an information-theoretic measure of surprise or uncertainty. Our aim in this article to establish whether the phenomenology of mood can likewise be analysed in terms of tending towards an optimal grip on a field of relevant action possibilities. We will argue that mood is a consequence of acting in the world with the aim of minimizing *expected* free energy. The key claim we set out to defend is that any agent that acts with the aim of minimizing expected free energy will have a feeling of how well or badly they are doing at tending towards an optimal grip. They will have what we will call a ‘feeling of grip’ that structures the possibilities they are ready to engage with over long time-scales, just as moods do.

In the first (Mood as a phenomenological structure of lived experience) section, we sketch a phenomenological account of mood (sometimes called ‘existential feeling’; Ratcliffe 2008). We show how mood structures the agent’s lived experience of the world by fixing the kinds of significance a situation has for them as they engage with the world. In the second (The feeling of grip) section, we revisit arguments developed in earlier work that the tendency towards an optimal grip can be thought of as arising out of an imperative to minimize free energy all living beings share in common. This allows us to frame the question which will occupy us in the remainder of the article of whether the phenomenology of mood can also be analysed in terms of tending towards an optimal grip. The third (How mood tunes prediction) section describes how mood can be understood in terms of momentum in minimizing expected free energy. Moods we suggest, summarize how the organism is faring overall in its predictive engagements, and tune expectations about how the organism will fare in the future. We suggest such an account of mood amounts to a redescription of the phenomenological account of mood in the terms of the FEP. In the fourth (Hopelessness and anhedonia in major depression) section, we show how our neurophenomenological account of mood might explain some key aspects of the lived experience (e.g. anhedonia and the loss of hope) that people can experience in major depression.

## Mood as a Phenomenological Structure of Lived Experience

In everyday lived experience, a person’s mood reflects how they find themselves in the world. The existence of a living being is never carefree. We always find ourselves in one mood or another because there is always some way in which the world matters to us. Even boredom and indifference are ways of finding ourselves situated in relation to the world. Heidegger’s term *Befindlichkeit* captures the phenomenology of mood well (Heidegger 1927/1962). The reflexive verb *befinden* is used to say how and where one finds oneself in relation to the world—it might for instance be used in responding to the question ‘How are you?’ (Colombetti 2014, 12). The situation in which we find ourselves can be pleasant, unpleasant, fascinating or boring, comforting or disturbed, safe or threatening and so on. These are descriptions of possible ways in which the world can matter, or be of significance to a person. A situation can show up as being significant to the person in these ways only because of the mood the person finds themselves in.

Moods are not internal subjective mental states that have as their cause external states of the environment. They arise out of our being in the world (Heidegger 1927/1962, 176). Imagine you are walking alone on a dark night in an unfamiliar part of town when you feel the presence of a stranger following you. The situation as a whole is all of a sudden experienced as one fraught with threat. Our emotion of fear is directed at the person following us, but only because the situation as a whole is experienced as threatening (Heidegger 1927/1962, 180). Threat in this example describes something of how you find yourself in the world. It is a mood that structures how you experience your current situation, and the kind of significance the situation as a whole takes on.

Ratcliffe offers a detailed analysis of the phenomenology of mood in terms of bodily feelings that situate an individual in the world, orienting them in a space of possibilities. For Ratcliffe moods fix the kinds of significance the world can be encountered as having, opening or closing the individual to specific possibilities (Ratcliffe 2008; c.f. Fuchs 2013). In the previous example, it is because you feel threatened that the world can be encountered by you as dangerous. Danger here is the kind of significance the situation can have because the situation you find yourself in is threatening. Ratcliffe’s talk of bodily feeling should be distinguished on the one hand from emotional states like fear, sadness or anger that involve some explicit appraisal or evaluation of the subject’s relation to the world. Emotions are sometimes felt in the body. They include what is sometimes called ‘core affect’ made up of arousal and valence components (Russell 2003). However, bodily feelings as Ratcliffe uses the term have a pre-reflective or background status—they make possible the full-blown emotional states a person can have. Think again of the example of the threatening situation—mood describes the way you find yourself situated in the world. Feeling threatened is a kind of concern on the basis of which one can experience an emotion such as fear of some particular thing or person (Heidegger 1962; Ratcliffe 2008). It is because our ways of caring about the world include being threatened that particular things can show up for us as being dangerous, and thus elicit an emotion of fear. Moods understood as pre-reflective background bodily feelings open us to a world of significant possibilities, a world with which we are involved, and in which we can feel more or less at home.

The notion of bodily feeling Ratcliffe uses to describe the phenomenology of mood should likewise be distinguished from bodily sensations such as itches, pains or tickles. (We thank an anonymous reviewer for emphasizing the importance of this distinction.) Bodily sensations are the more or less subtle changes sensed at specific locations in the body from the inside. Bodily feelings however need not be localized bodily experience but can consist in a ‘general sensitivity to the world’ (Slaby 2008). Moreover, bodily feelings need not be discrete episodes with a more or less well-defined, dateable beginning and end. They can arise in the course of our wider activities, structuring our overall style of engagement with the world. Bodily sensations can sometimes structure the person’s sense of what is possible. Think of how pain for instance can infuse your overall experience of your situation, shrinking your openness to the world and the practical possibilities it offers. People that suffer with migraine for instance, sometimes say all they can do is hide away in a dark room. The distinction between bodily feelings and sensations is thus not always sharp. It is nevertheless a distinction it is important to observe if we are to use the notion of bodily feeling to understand the phenomenology of mood as Ratcliffe proposes. Many sensations are localized bodily episodes like itches that occur without the sensation structuring the person’s overall sense of the kinds of possibilities the world offers. Bodily sensations can occur that do not incorporate any world-directedness beyond what the person senses in their body. Moods by contrast arise out of a person’s being in the world, making manifest how a person is faring in their engagement with the world. Lauren Freeman describes the phenomenology of mood well when she writes: ‘We are in moods with the entirety of our being, and out of our moods the world as a whole is disclosed to us’ (Freeman 2014, 453).

Moods have been analysed in philosophy as ‘generalized emotions’ that lack a clear focus because they are directed towards the world as a whole, not towards particular objects (see e.g. Solomon 1993, 71). The difference between mood and other emotions is that moods provide a general colouring or tone to emotional experience. In psychology moods are typically understood as long-lasting, internal states of subjects that differ from emotions only in their duration and lack of a specific object they are directed towards (see e.g. Ekman and Davidson 1994, and for critical discussion, Freeman 2014). Such a conflation of mood with emotion cannot be correct if mood is what makes it possible for things to be of emotional significance to us. Without mood there would be no emotion. If the world lacks practical significance for a person for instance, as it does in some experiences of major depression, the emotions of hope or worry about some practical project and its outcome no longer make sense. The depressed person literally experiences a world without hope in which no projects call out as worth pursuing (Ratcliffe 2015).

Mood we suggest structures lived experience by entailing an anticipation-fulfilment structure for lived experience. Think of the feeling of hope that takes root after an interview for a big project grant goes really well, and one comes to expect a positive outcome. Then the disappointing news is received that the application was unsuccessful. Binswanger describes the experience well when he writes how ‘the world—in one stroke—becomes “different”’ (Binswanger 1975, 222–3). The meaningful possibilities one anticipated as on the horizon, immediately fall away. As a further example familiar to many of us, consider Ractliffe’s example of feeling at ease in teaching a class one has prepared carefully in advance. He describes how one experiences the class situation as a whole as the ‘confident, unproblematic anticipation and fulfilment of various significant possibilities’ (Ratcliffe 2017, 164). One’s engagement with the class is characterized by a ‘diffuse feeling of ease or confidence’ (*Ibid.*). Ratcliffe’s example is an illustration of a skill-based understanding we bring to every encounter with the world on the basis of which we have an experience of belonging, or being at home in the world.

In the next section, we begin the work of making sense of this phenomenological conception of mood in neurobiological terms. We aim to show how the anticipation-fulfilment structure of lived experience can be mapped onto the anticipatory dynamics of free-energy minimizing systems that tend towards an optimal grip in their engagements with the world. The question we take up in the rest of our article is whether the role of mood in shaping the anticipatory structure of lived experience can likewise be understood in terms of the dynamics of free-energy minimization.

## The Feeling of Grip

The FEP posits that all systems that maintain their organization in their exchanges with a dynamic environment will minimize their own free energy. The principle was formulated by Karl Friston to understand the organizational properties of complex biological systems, from plants and single cells all the way up to humans, and the social networks they form with each other (Friston and Stephan, 2007; Friston 2010, 2013; Allen and Friston 2018). What these biological processes all share in common is that they show *self-organizing* behaviour. They are open systems in which global order arises through the coupling of the system’s internal dynamics to the external dynamics of the system’s econiche. Second, and relatedly, all of these biological processes are adaptive systems that maintain their organization when perturbed from the outside. FEP purports to identify the organizational properties a complex adaptive system must have if it is to preserve its organization for an extended and prolonged period of time in a dynamic environment. What behaviours must the system select—how must it act—if it is to continue to exist, and stay away from catastrophic phase transitions (destructive changes in its organization)? In this section, we revisit our ecological-enactive interpretation of the FEP with the aim of bringing out how FEP calls into question any dualistic separation of bodily feeling from life. (Our ecological-enactive interpretation of FEP is developed in more detail in earlier articles co-authored with Jelle Bruineberg—see Bruineberg & Rietveld 2014, Bruineberg, Kiverstein & Rietveld 2018.) FEP as we will interpret it implies that complex adaptive systems must be agents with their own concernful point of view on the world. Feelings (and we will eventually argue, mood) grow smoothly out of such concern.

### 2.1. The FEP: an ecological-enactive reading

The FEP takes as its starting point the observation that any complex adaptive system must actively regulate its behaviour so as to remain in a dissipative, far from thermodynamic equilibrium steady state (Friston and Stephan 2007). Complex adaptive systems are by definition, systems that are able to stay away from thermodynamic equilibrium—a state of a physical system in which there is no exchange of matter and energy either within the system, or with the environment. Thermodynamic equilibrium means death for living systems: a system isolated from external influence will, in accordance with the second law, increase in disorder or entropy over time. Complex adaptive systems keep entropy to a minimum by for instance finding the energy they need to fuel their actions. To endure for any period of time, living systems must at the same time negotiate a changing environment so to avoid destructive phase transitions. They must for instance avoid extremes of temperature or pressure that threaten their continued integrity. More generally, they must act selectively on the environment in ways that fit with change in their surroundings.

The FEP states that the actions any complex adaptive system should select are those that minimize surprising or improbable sensory exchanges with the environment. The internal states of a complex adaptive system can be described in terms of an attractor landscape—a set of attractor states the system tends to revisit regularly over time under a wide range of initial conditions (Friston 2012). The set of attractor states for the system will be internal states that are highly probable given the system’s phenotype and the kind of niche it inhabits. In the FEP, the internal states of the system are said to be a ‘model’ of the system’s ecological niche by means of which the system can steer its actions. The purpose of the model is to anticipate perturbations imposed by the ecological niche so that the system can adapt its actions appropriately. For this reason, we have described the generative model as a ‘system of anticipations’ (see (Bruineberg & Rietveld 2019). (We should note this is perhaps not the standard way of understanding the generative model. The generative model is standardly understood as functioning like a map that encodes statistical regularities on the basis of which the organism can infer the hidden causes of its sensory signals (Gladziejewski 2016; Kiefer and Hohwy 2017). (See Ramstead et al. (2019) for a critique of this standard understanding of the generative model, and an enactive reading of ‘model’ talk in line with the one we assume in this article.) The model in steering the system’s action generates exteroceptive, proprioceptive and interoceptive sensory states that form a narrow region of the state space that defines the possible sensory states of the system. Sensory states that fall within this region are unsurprising and expected, while sensory states that fall outside of this narrow region are improbable for the system, and thus are surprising, or unexpected. To remain well adapted to its environment, the system should avoid sensory exchanges that are surprising, and sample the environment for sensory outcomes that are expected. Organisms however have no tractable way of calculating the surprise value (or improbability) of their sensory states. The FEP says organisms get round this problem by using free energy as an upper bound on surprise. Free energy is always greater than surprise. Thus by keeping free energy to a minimum biological systems will also succeed in minimizing surprise (Friston 2010, 2012).

The FEP in a nutshell ‘states that all the quantities that can change (i.e. that are a part of the system), will change to minimize free energy’ (Friston and Stephan 2007). These quantities are the internal dynamics (e.g. the parameters of the agent’s internal model biologically realized as weights and biases in the system’s neural dynamics if it has a brain), and the external dynamics in the environment which the agent intervenes in through its actions. The result of minimizing the free energy of its internal dynamics is that the agent’s internal dynamics will be well adapted to causal regularities in the environment. The model can be used to steer the agent’s actions because the outcomes that are anticipated based on the model are ones that closely approximate what happens when the agent acts. The organism will thereby tend on average to avoid surprising and improbable sensory states. By minimizing the free energy of its internal dynamics, the system will also keep surprise to a limit.

The second quantity that can change so as to minimize free energy is the external dynamics that describe the agent’s interaction with its environment. We take the external dynamics to describe the dynamics of the agent’s eco-niche. Through its actions the agent can change the structure of the environment, bringing about sensory states that match those the agent expects given its internal dynamics. The agent will selectively sample the space of possible sensory inputs for those sensory outcomes it expects to encounter. The sensory outcomes the agent expects will be fixed by the agent’s phenotype and the ecological niche it grows up in. So long as the agent succeeds in fulfilling its expectations through its actions, it will typically find itself in sensory states that are unsurprising, avoiding improbable sensory encounters that are a threat to its viability. By minimizing the free energy of its external dynamics, the agent will thereby bound the surprise value of its sensory states.

The FEP posits that so long as the internal and external dynamics change over time to minimize free energy, the result will be that the organism will remain well adapted to its niche. We have seen above how the FEP states how a living system *should* act if it is to remain viable in a changing environment. The actions the organism should select if it is to minimize free energy are the actions likely to lead the organism to remain well adapted in its engagement with the ecological niche, and thus to flourish. Free energy thus quantifies how well or badly the organism is doing in its engagement with the environment. Organisms should (if they are to succeed in minimizing free energy over time) self-regulate their coupling with the environment. They should continue on a promising path when all is going well, or when things are going better than expected. They should shift to doing something else when things are not going as well as expected. If they are to minimize free energy, organisms should develop an internal dynamics geared to steering them in the direction of flourishing.

### 2.2. The tendency towards an optimal grip

So far in this section, we have described how living systems should actively regulate their engagement with the world so as to minimize free energy because doing so will maximize the probability of their own flourishing in their niche. Crucially, a system that modulates its engagement with the environment according to its sense of how well it is faring can be said to have an ‘inner life’—a point of view on its environment relative to which it can make distinctions between doing better or worse, well or badly. The FEP thus calls into question any separation of the inner life of the organism from its biological organization. We have seen above how any system that succeeds in minimizing free energy will regulate its engagement with its niche on the basis of its own inner sense of how it is faring. Nor does it make sense to separate the behaviour of the organism from its sense of how well or badly it is attuned to what it cares about in its environment. The sensory states the organism aims to bring about through its actions correct for deviations from the organism’s own flourishing in its niche. This state of flourishing can be thought of as an optimum for the organism. *The organism senses its deviation from this optimum* and acts to move closer towards an optimal relation to its ecological niche. We call this dynamic ‘the tendency towards an optimal grip’, drawing upon Merleau-Ponty’s notion of *maximal prise* (Merleau-Ponty 1962/2012).

Consider as an illustration, Merleau-Ponty’s description of the relation the football player takes up to the other players and the football field in a game of football. Merleau-Ponty describes the football field as ‘pervaded by lines of force (the ‘yard lines’; those which demarcate the ‘penalty area’) and articulated in sectors (for example the ‘openings’ between the adversaries) which call for a certain mode of action’ (Merleau-Ponty 1942/1963, 168). The different regions of the pitch and the other players are marked out for the player as inviting specific actions more or less appropriate to what is happening in the game as it unfolds. There are a near open-ended number of possible actions available to the player. Of the possible actions the player could perform, some stand out as to be done here and now as the game unfolds. Others concern the directing of the future course of the game as an attack builds. Still other actions are just not relevant possibilities at all in the player’s current situation, such as lying down on the grass in the midst of the game to take a nap.

We suggest it is based on their sense of how they are currently faring as a skilled player that affordances stand out as inviting in the game. We borrow the term ‘affordance’ from the ecological psychologist James Gibson using the term for the possibilities for action the environment makes available (see Gibson 1979, chapter 8). To be more precise, we define affordances as relations between aspects of the socio-material environment in flux and the abilities available in a form of life (Rietveld & Kiverstein 2014; Van Dijk & Rietveld 2016). So we use the term ‘affordance’ in a broad sense—the affordances of the human environment are as rich and extensive as the skills and abilities available to humans. We call relevant affordances that stand out as calling for action, ‘invitations’ (Dreyfus and Kelly 2007; Withagen *et al.* 2012). The skills the player has developed give them a feel for how well or badly things are going in the game, both at a given moment in time and looking ahead into the future (Rietveld 2008). They have an immediate felt appreciation for what would be required for things to go better. It is on the basis of this feel for the game that the affordances unfolding in the game stand out as inviting, calling to them to take a certain course of action. In responding to inviting affordances agents tend towards an optimal grip; they can be ‘moved to improve’ by these invitations (Rietveld 2008). Certain affordances stand out as inviting because the agent deviates from an optimal relation of equilibrium with the environment. Affordances are inviting when they move the agent closer towards equilibrium—when they enable the agent to improve its grip on the possibilities that matter to them as a skilled agent.

Humans are skilled at acting in many different contexts and situations. They have grown into and become skilled participants in a multiplicity of different practices in their everyday life. They thus have a multiplicity of different (and sometimes conflicting) cares and concerns that feed into their sensitivity to how they are faring in their practical engagement with the word. The person will thus always be readying themselves for acting on several relevant affordances. The multiple relevant affordances the individual is ready to engage with form a structured field in dynamic flux. In acting to minimize free energy, the skilled individual will be ready to act in ways that are responsive to a whole field of relevant affordances.

The question we take up in the remainder of our article is whether the tendency towards an optimal grip on a field of relevant affordances can account for the phenomenology of mood. Can we use the tendency towards an optimal grip to explain how mood can structure the person’s sense of what is significant in the situation they find themselves in? Anticipation we have been arguing should be thought of in terms of free-energy minimization. The significance the individual experiences their situation as having should therefore fall naturally out of an agent that is sensitive to how well or badly they are doing in minimizing free energy. We explore how this might work in the remainder of our article.

## How Mood Tunes Prediction

We have seen in the first (Mood as a phenomenological structure of lived experience) section how the mood a person finds themselves in structures the kinds of significance they experience in a given situation. Is the notion of ‘inviting affordances’ sufficiently broad in its application to cover the many kinds of significance the individual can experience? Ratcliffe has argued it is not. He claims that the notion of affordance is too coarse-grained to capture the different ways in which a situation can appear meaningful to an individual (Ratcliffe 2015, 61; c.f. Dings 2018; Ratcliffe and Broome, forthcoming). A situation can be exciting or boring, comforting in its familiarity or disturbing in its strangeness. A situation can be of interpersonal significance offering up possibilities for conversation, and friendship, pride or shame. Ratcliffe has argued that such differences in the significance a situation can have cannot be understood in terms of the way in which affordances can invite action. Ratcliffe’s critique stems from his restricting his focus to the invitation of a single affordance, and the attractive or repelling quality of an individual affordance. We would argue however that inviting affordances do not have significance taken in isolation from the structure of the field of relevant affordances as a whole. It is to the structure of the field as a whole that we should look for an account of the kinds of significance Ratcliffe takes moods to disclose.

For the person experiencing hopefulness, for instance, the field will have a structure with temporal depth, in which the future offers the promise of many inviting possibilities. For a person experiencing boredom, the field will be structured so that the immediate situation offers nothing of interest. We’ve suggested that the field of relevant affordances has the structure it does because of the individual’s skills and their immediate felt sense of how well things are going in their dynamic engagement with the world. We propose to think of mood as giving an individual a feel for how well or badly they are gripping to the field as a whole. On the basis of this feeling, the situation as a whole is experienced as having a particular significance.

### 3.1. Error dynamics track grip

When you ask a person how their day is going they will likely answer by giving a general overall sense of how they are doing. They say they are doing well or badly or perhaps just fine. They are indicating something about how they find themselves in the world given what matters to them. We suggest this overall sense of how well or badly things are going is based on expectations of how free energy is likely to change over time. It has recently been proposed that the positive or negative valence of emotions can be understood in terms of the rate of change in free energy (Joffily and Corricelli 2013; for a related proposal see Van de Cruys 2017; Kiverstein *et al*. 2019; Miller et al. 2020). The negative valence of fear maps onto free energy increasing faster over time. The positive valence of hope maps onto free energy decreasing at a fast rate. We will refer to the change in free energy over time as ‘error dynamics’ because free energy is a measure of the mismatch between the sensory states the agent expects given the generative model it embodies, and the sensory states it comes to occupy. Whenever there is a mismatch detected and thus a change in free energy, this is because the agent’s expectations are in error. (When we use the term ‘error’, we do not mean this term to be understood in a semantic sense. It is common in the literature on the FEP to interpret the generative model as encoding prior beliefs with semantic content. Error can thus be interpreted as a consequence of the generative model failing to represent the hidden external causes of sensory input correctly. We suggest by contrast an understanding of error in terms of pragmatic success—error has the consequence that the agent fails to adapt its actions adequately to its econiche.) Change in free energy over time can thus be thought of in terms of error dynamics—how fast or slow error increases or decreases over time.

The FEP states that the generative model the organism embodies aims to keep free energy to a minimum over time. Error dynamics can thus be thought of as providing feedback for the organism about the quality of the generative model it embodies. This feedback informs the organism of how well or badly the model is doing at adapting the organism to its econiche. More specifically, the change in the quantity of free energy can be used by the agent to track volatility or uncertainty about the temporal evolution of states in the environment (Joffily and Coricelli 2013). Free energy increases or decreases with environmental volatility. The more volatility there is estimated to be in the world, the more uncertain the agent should be about acting. Thus error dynamics can be used as a proxy for uncertainty about the outcome of actions. Fear for instance is experienced by an agent in a situation in which free energy is increasing at an accelerating rate. Increasing free energy is equivalent to accumulating surprise. It signifies that the sensory evidence the agent has sampled fails to match what the agent anticipates given the model it embodies. The agent should not rely on such a model to steer its actions—it cannot be confident that the actions it selects will lead to the sensory outcomes it prefers. Now contrast this with the situation in which the agent is hopeful because free energy is being reduced fast. The agent can in this situation be confident that the actions it selects will lead to the preferred outcomes it expects.

Free-energy minimizing agents should not only care about uncertainty in the present but should in addition make use of their past experiences to keep track of *future or expected free energy*. We have seen how free-energy minimizing agents expect to do well and flourish in their niche. They act in ways that aim to fulfil this expectation. They face the challenge of how to transition from their current situation at time *t*_0_ (e.g. hunger) to their preferred state at some future time *t*_1_ (e.g. digesting a nutritious meal). We have seen above how the multiple affordances stand out as inviting are the affordances the agent anticipates will lead them from their current situation (at *t*_0_) to the sensory outcome in the future (at *t*_1_) they expect. The agent’s present situation provides sensory evidence that favours to differing degrees, a selection of the action possibilities available to them. The agent must select affordances that minimize expected free energy—the difference between the sensory states the agents expects to sample in the future (i.e. those that are associated with a state of flourishing or well-being, e.g. being well-fed), and those attainable from their current situation. The affordances that minimize expected free energy should stand out as relevant to the agent and as inviting action.

Following Joffily and Coricelli (2013), we suggest that error dynamics can be used as a measure of the likely performance of an action policy. Joffily and Coricelli (2013) base their hypothesis on the simulation of an agent that learns to maximize reward in playing against a one-armed bandit slot machine under varying conditions of volatility. One agent explicitly estimates the volatility of environmental states (the probability of reward), while the other agent relies on what we have called error dynamics—the change in free energy over time. They show (among other things) how the agent that uses error dynamics to track volatility can not only match the performance of an agent that explicitly estimates volatility but can also out-perform such an agent when shifting from conditions of low to high volatility. They write ‘With the addition of emotional valence to the model, the agent becomes even more reactive and is able to track fast changes in the environment’ (p. 7).) Insofar as the agent is aiming at minimizing future or expected free energy, they must somehow keep track of whether the free energy of their current sensory states and the free energy of their future sensory states increases at a faster or slower rate than expected as a consequence of their actions. It is an important and (to our knowledge) open question how the tracking of error dynamics might be neurobiologically implemented. Work on active inference suggests that neurotransmitters such as dopamine will do part of this work (Fitzgerald *et al.* 2014; Friston *et al.* 2012, 2014; Schwartenbeck *et al.* 2015a,b). Dopaminergic midbrain regions are widely accepted to play a role in tracking reward prediction errors (see e.g. Schultz *et al.* 1997)—they track whether the outcomes of the individual’s actions were better or worse than expected. Thus when an animal receives more food than expected this is strongly correlated with dopaminergic change in the nucleus accumbens (Hart *et al.* 2014). A similar response was found in humans when they performed better than expected in a financial reward task (Rutledge *et al.* 2010). In work on active inference, the dopamine system is taken to be a part of a complex dynamical system that optimizes precision expectations through learning. (This process of optimization is given a neurobiological characterization in terms of optimizing the sensitivity of post-synaptic gain of cells. Phasic discharges in the dopamine system are hypothesized to signal error in precision expectations. Tonic discharge of dopamine is hypothesized to influence the post-synaptic gain on such error signals leading to an update of precision expectations (Friston 2012, 276).) We have suggested above that active inference is the process of selecting the relevant affordances that are expected to minimize the mismatch between current and future sensory inputs the agent expects to occupy so long as it is flourishing. Dopaminergic discharges weigh the agent’s confidence in relevant affordances given the agent’s skills and abilities (Friston *et al*., 2012; Miller, Kiverstein & Rietveld 2020; Kiverstein, Rietveld, Slagter & Denys 2019; Linson, Clark, Ramamoorthy, & Friston 2018).

We are suggesting the dopaminergic system is part of a complex system sensitive to changes in the rate of error reduction. Weighing the precision of relevant affordances is work best done in part through keeping track of error dynamics (Miller *et al.* 2020). Information about rate of change in error reduction is valuable feedback that can be used to fine-tune precision estimations. If the dopamine system is implicated in the process of precision estimation (as is suggested by Friston *et al.* 2012; Fitzgerald *et al.* 2014; Schwartenbeck *et al.* 2015a,b), dopaminergic neurons should keep track of error dynamics. (We focus above on the role of the dopamine system in enabling the person’s sensitivity to error dynamics because the precision estimation has been shown to map onto reward learning systems in the brain (see e.g. Fitzgerald *et al.* 2014; Schwartenbeck *et al.* 2015a,b). The recent literature on active inference suggests however that there may be multiple ascending neuromodulatory systems involved in estimating precision. Parr and Friston (2017) hypothesize that cholinergic projections to the cortex may track precision of expected outcomes given states of world, while noradrenergic systems track the precision of state transitions or unexpected uncertainty arising from environmental volatility.)

By using error dynamics to weigh the precision of relevant affordances, the agent can maximize the probability that the sensory states it brings about through its actions are ones that minimize *expected* free energy. To see this, consider how to minimize expected free energy the agent must maximize the predictive success of the model it instantiates (c.f. Phillips 2013). It is only if the agent can accurately and precisely forecast the future sensory consequences of its actions that the agent will succeed in selecting the relevant affordances most likely to minimize expected free energy. (See Hesp and colleagues’ (preprint) for a detailed computational analysis of the role of valence in the process of selecting actions that minimise expected free energy. Their account of affective valence complements well the account of mood we develop in this article.) Using error dynamics as feedback will therefore help to maximize the quality of the generative model and its predictions. It will ensure that the agent continually makes progress in developing its skills, and actively seeks out sensory states that are not so complex as to be unpredictable, nor so simple as to be fully predictable no longer allowing the agent to learn anything. (Evidence that this is indeed the strategy free-energy minimizing agents deploy comes from research on learning progress in developmental psychology and robotics. Thus Kidd *et al.* (2012) show that 7- to 8-month infants show a preference for sequence of events that are neither too predictable as to bore them, nor too complex so that they are unable to predict them. They were most likely to look away when complexity was either too high or too low, and prefer to look at sequences that are novel enough to hold their interest. The enjoyment of novelty helps the infant to continually make progress in learning, allowing for the learning of a generative model able to manage a dynamical environment over increasingly longer periods of time. This hypothesis is borne out by work in epigenetic robotics in which progress in learning is associated with an intrinsic reward, and the robotic agent is motivated to act in ways that maximize this reward (Oudeyer and Smith 2016). For further discussion, see Kiverstein *et al*. 2019.) An agent that aims to continually make progress in its predictions will tend to be drawn into action by relevant affordances that maximize the probability of its continually improving its grip on the possibilities that matter. By maximizing the predictive success of its model, the agent will thus maximize the probability of its own flourishing (Kiverstein *et al*. 2019).

### 3.2. Mood as momentum in free-energy reduction

We suggest that positive and negative moods can be thought of as a reflection of the overall *momentum* or expected direction in error dynamics. (We base this proposal on work by Eldar *et al.* (2016). They frame their hypothesis in terms of reward learning but reward can be shown to map onto expected sensory states using the FEP as we briefly explained above. (For a more detailed account see e.g. Friston *et al.* 2009). James Clark in a co-authored paper with Friston has proposed an account of mood that converges with the one we are outlining (Clark *et al.* 2018). Like us, they propose to understand mood in terms of expected free energy. Clark *et al.* suggest that emotions track uncertainty about the consequences of action—the precision with which motor and physiological states consequent on action can be predicted. Moods they argue are expectations about uncertainty that determine how emotions change over time. Thus, the low mood people experience in major depression they conceptualize as certainty that the person will encounter an uncertain, volatile environment. In anxiety disorders (which they distinguish from major depression), the person expects with low confidence to encounter a volatile and unpredictable environment. Finally, Clark *et al.* show how in mania a person may expect with high confidence positive (expected rewarding) outcomes of their actions. We are grateful to an anonymous reviewer for drawing our attention to the work of Clark *et al.*) Moods are expectations of rate of change in free energy. They are projections for the agent of how well or badly their activities are likely to go in the future.

By ‘momentum’ we mean the expected rate of error reduction over time. We use ‘momentum’ to refer to a general trend in free-energy reduction. Given their past experience, can an agent expect a future in which free energy will be reduced at an accelerating or at a decelerating rate? A positive mood like happiness depends not only on things going well, but on them going better than you had expected (Rutledge *et al.* 2014). Positive mood consists in an expectation of positive momentum in expected free-energy minimization. The agent expects that given its current situation free energy will decrease at an accelerating rate as a consequence of their actions. This biases upward their estimation of the probability of relevant affordances leading to preferred sensory outcomes. This upward biasing of expectations makes perfect sense in a natural world with all kinds of intermeshing regularities. For example, rewards are commonly interconnected, and often occur contiguously. A good rainy season means more food all around; spring means more food, while winter means food scarcity (Eldar *et al.* 2016). There is thus an adaptive advantage to allowing local successes and failures to help tune more global expectations about rewards. If for example, we find berries on a few bushes in a row, perhaps this means spring has arrived. In that case, we may benefit from updating our expectations about future berry finding in the area as a whole (not just on individual bushes).

Positive mood provides feedback for the organism of the overall, global trend in how it is faring, and whether or not the organism can expect to experience good times going forward into the future. A few experiences of positive (i.e. expected) outcomes in a row will lift mood, which if all goes well should steer the organism in the direction of expecting free energy to continue to decrease in the future. Similarly when one does not have a good grip on a given situation, perhaps because the situation is too complex, momentum in free-energy reduction will be negative. One will start to expect more failures and further increasing disattunement. In this case, negative mood provides feedback indicating the problematic overall negative trend in momentum. By tracking rate of change in free-energy reduction mood provides feedback that allows an agent to take into account general trends and the ‘impact of multiple environmental factors’ without having to calculate the individual impact of each factor (Eldar *et al.* 2016).

Given the structuring of expectations by moods , a rigidifying of either positive or negative mood will have detrimental consequences for the person’s well-being. A rigid positive mood will sooner or later lead an agent to fail to anticipate which affordances to engage to bring about preferred outcomes. Negative mood can enable an agent to engage flexibly and adaptively when things change by bringing expectations of opportunities for free-energy reduction back in line with reality. At some point, all the berries will have been consumed from the bushes. Thus, to continue to seek berries in a particular location will no longer serve the agent well. Negative moods signal negative momentum—the agent expects acceleration in the increase of free energy as consequence of their actions, leading to the biasing downwards of estimations of the precision of an inviting affordance (e.g. searching for berries on these very bushes). When opportunities to improve grip begin to diminish in this way, the ensuing negative mood allows our expectations to quickly tune to the unreliability of relevant inviting affordances for improving grip in the current circumstances.

So far we have been describing how mood may bias individuals to weigh precision in ways that maximize opportunities for the minimization of expected free energy. This would mean that when in a positive mood individuals should be more sensitive to and on the lookout for affordances that predict positive momentum—an acceleration in free-energy reduction. They will sample the environment for opportunities that confirm this expectation of a positive trend, predicting in extreme cases an overall state of flourishing in the future. This biasing of attention by positive mood in a dynamically changing environment will however inevitably lead to surprise. Joffily and Corricelli (2013) suggest it is a signature feature of positive mood understood in terms of error dynamics, that it will tend in the end to lead a person to overlook important changes in the environment (p. 4). The effect of this failure to attend to change will be an accumulation of error which will lead to negative affect, and a consequent swing to negative mood—an expectation of deceleration in the reduction of free energy. If the rate of change in free energy is greater than the individual expects (i.e. if there is a deceleration in free-energy reduction), this should grab their attention. They should begin to explore their environment on the lookout for opportunities to do better. As the person begins to fare better and free energy begins once again to decrease, this should feel good. Eventually, this will result in a swing to a positive mood when momentum takes a turn in the direction of acceleration in free-energy reduction.

Indeed, we suggest that a positive mood should be associated with a higher learning rate (i.e. high estimates of precision) for negative outcomes. By ‘negative outcomes’ we mean breaches in expected positive rate of change or acceleration (i.e. violation of expected continued momentum; c.f. Eldar *et al.* 2016, 7). Positive mood has been shown to dominate in healthy individuals (Diener and Diener 1996). Healthy individuals typically enjoy what is referred to as an ‘optimism bias’ (Sharot *et al*. 2016). The reason for this, we speculate, lies in part in the dynamic we have just described. Positive mood induces in individuals the expectation of continued trend of acceleration in free-energy reduction. At the same time, they fail to attend to changes in their circumstances which have the consequence that free energy begins to accumulate and momentum shifts downwards. Learning from negative momentum means the agent will be quick to recalibrate their expectations in a downward direction. Once their expectation for acceleration in free-energy reduction is brought down, it will become easier for them to find their way to opportunities for meeting those expectations. The result of this will be the agent begins to do better than expected and returns once again to a positive mood. Thus they will tend to spend more of their lives in a positive than negative mood.

To illustrate this virtuous circle induced by positive mood, suppose that a person expects to be an A student. They feel confident in their ability to learn. However, when they arrive at university they find they are now a C student. This would have the consequence that each time they encounter the same event (receiving a C on another paper assignment) this would result in negative surprise (feedback that they have done worse than the expected A grade). They start to feel bad, and this leads them to adjust their expectations of their own performance. Negative mood that arises from doing worse than expected (i.e. failing to fulfil their optimistic expectation of receiving an A) should, we hypothesize, lead to progress in learning. They start to expect to do badly, which in turn allows them to learn new strategies for improving their performance. This will allow them to benefit from incremental improvements relative to their actual (not just expected) skill level. They might for instance develop new action policies for studying. This will, if all goes well, in turn generate positive surprises, and thus improve their mood as their grades incrementally improve from the C they have begun to expect back up towards the performance of an A grade student.

When moods are functioning well in a free-energy minimizing regime, the strength and duration of the mood will fluctuate with rising and falling opportunities for minimizing expected free energy. The result of this tracking of momentum will be a tuning of a model that enables the agent to continuously tend towards an optimal grip on the field of relevant affordances as a whole. When things are going well, the agent will tend to continue on the same path. When things begin to take a turn for the worse, so also will their mood. Thus, the individual will be moved to do things differently, or even switch to doing something else, which will if things go well help to restore their fortunes.

However, changes in mood can only contribute to this improved grip when they help to shift upward or downward the agent’s precision estimations (i.e. the confidence the agent has that a relevant affordance is likely to lead to its expected state of flourishing). Precision estimates should ideally track the probability of an affordance leading to outcomes the agent expects. The agent’s confidence should march in step with the actual possibilities for minimizing expected free energy, or catch-up when such estimations fall behind (i.e. when the agent does worse or better than expected). As we have noted above, positive moods should persist only as long as the general trend is one in which the environment is providing better than expected opportunities to flourish. To offer an extreme example, research shows that lottery winners only enjoy a brief period of elation before returning to basically the same sense of wellbeing they had before they won (Brickman and Campbell 1971; Brickman *et al.* 1978; c.f. Van de Cruys 2017).

Not adjusting confidence when one’s circumstances take a turn for the worse can lead to serious dysfunction. For example, if an activity (for instance using a drug) repeatedly does better than expected at reducing free energy, then this activity will begin to stand out as highly inviting and salient. The activity will over time tend to be repeated more and more. This is what happens in substance addiction, where for example opioids act on the precision estimation circuitry in the midbrain so as to reinforce drug-seeking action policies (Schwartenbeck *et al.* 2015a; Miller *et al.* 2020). The result is that the drugs of addiction attract the user in increasingly powerful ways, while other opportunities for reward (social relationships, career, nutritious food, *etc*.) are increasingly ignored to the detriment of the organism. People grow up to expect a good relationship with family, a successful career, and operate with a biological expectation for food when hungry. Addiction leads to all of these expectations being frustrated, and thus to a pervasive build-up of free energy. Yet despite these negative outcomes, the affordances the use of the drug offers continue to be estimated as a good bet for minimizing expected free energy. It is this rigidification of the agent’s drug-using action policy, and the failure of the precision that is given to this policy to keep up with the overall downward momentum in the addict’s fortunes that can make addiction pathological. We can think of this in terms of the shrinkage of the field of relevant affordances to only those affordances that relate to the use of the addictive substance.

Major depression can arise when an agent operates with overly rigid expectations of positive momentum in free-energy reduction. The agent fails to correct its expectations when momentum turns negative, as we will discuss in more detail in the next section. The failure to recalibrate their expectations leads to a negative, and vicious circle of the opposite kind to that seen in healthy individuals that tend to consistently return to a positive mood. The depressed individual continues to expect an unrealistic positive momentum of free-energy reduction. They do so because unlike healthy individuals they exhibit a low learning rate for negative momentum. As long as they do not adjust their expectations of free-energy reduction downward, the world will continue to frustrate their efforts at acting in ways that allow them to flourish. Failing to flourish will become unsurprising to them. Such an agent will soon find themselves locked into a vicious cycle in which nothing they do contributes to bettering their situation. If the agent continues to meet the world with such expectations, no affordances will call to them as likely to lead to good outcomes. They will experience a field of relevant affordances in which everything is flat and nothing in particular stands out as worth doing either in the present, or going into the future.

## Hopelessness and Anhedonia in Major Depression

Solomon in writing about his first-person experience of major depression tells us: ‘the first thing that goes is happiness. You cannot gain pleasure from anything’ (Solomon 2002, 19). The person experiencing major depression expects that nothing good could happen. Affordances that would previously have been inviting to the agent (e.g. succeeding at work, eating a fine meal) begin to appear less inviting, and so cease to draw the organism into action in the same way.

The persistent low mood associated with major depression we propose to understand in terms of negative momentum in expected free-energy minimization. We suggest low mood persists and rigidifies because the agent exhibits the opposite profile to healthy individuals—they exhibit a low learning rate for negative momentum in free-energy reduction. Evidence of low learning rate for negative momentum in depressed individuals comes from work showing that low serotonin makes people insensitive to negative outcomes (Bari *et al.* 2010; Eldar *et al.* 2016). This low learning rate results in overly rigid optimistic expectations. The person acts with expectations of free-energy reduction the world consistently fails to meet. An anonymous reviewer objected we must be mistaken in attributing overly optimistic expectations to people with major depression. The reviewer rightly observed that people with major depression show a pathological under confidence in the predictions of the outcomes of their actions coupled with a deep lack of self-worth. We are suggesting however that it is their harbouring of overly optimistic expectations that leads them to lack self-worth, and to have low confidence in their own abilities. For example, the person may expect parents that love and care for them but encounter only parental abuse and violence. The repeated frustration of their expectations of a nurturing home environment would lead to persistent and recurring error, and thus to low mood. Moreover, the frustration of this expectation for a stable and safe home environment is one they cannot change through their actions. Nothing they do seems to make any difference. Thus operating with what turns out to be an overly optimistic expectation will lead to an increase in free energy over time, and thus to low mood. While their low mood persists, they will continue to expect only uncertainty and thus to lack confidence in themselves and in the opportunities for flourishing the world offers (c.f. Clark *et al.* 2018). Some of these expectations may come from their phenotype and concern biological imperative like living in a safe and nurturing social environment that are frustrated by for example parental abuse and neglect, or growing up in a warzone. Normally this breaching of expectations would eventually lead to a recalibration—to an expectation of deceleration in free-energy reduction. But we suggest that because of low learning rate for negative momentum in free energy reduction this recalibration fails to occur. They do not adjust their expectations for positive momentum which means they don’t find their way to opportunities to do better in the way that healthy individuals do. Typically if an agent expects free energy to increase as a result of their actions, they will explore for alternative possibilities they can instead rely upon to lead them towards the outcomes they desire. When they find such affordances they will get the feedback that things have gone better than expected, and this improvement in fortune should over time translate into an improvement in mood. Recall again our example of the student above who had adapted his or her expectations so as to better make progress in their learning. But for the depressed individual none of this takes place. Instead they meet the world expecting only negative, unpredictable outcomes—they ready themselves for a world that is threatening to their well-being. (We see this in the physiology of the person in the hyperactivity of the hypothalamic-pituitary-adrenal axis—the body stress response—and in proinflammatory immune activity that produce sickness behaviours aimed at reducing energy expenditure (Barrett *et al.* 2016).)

The source of the problem is low learning rate for negative momentum. The failure to update unrealistic expectations for free-energy reduction leads to rigidification of low mood. This has the consequence that the person’s precision estimates fail to march in step with the natural rises and falls in free energy that are a part of every individual’s engagement with a dynamic environment. Their persistent low mood means they fail to anticipate where the opportunities for improving grip are to be found. At this point an enduring feedback loop may emerge, driven by top-down expectations, whereby the organism begins to sample the environment in ways that confirms their depressed outlook. There is an important temporal progression to take note of here in the formation of this feedback loop. Low mood initially results from violations of expectation of positive momentum in free-energy reduction. Over time, the person comes to expect violations of such optimistic expectations—they come to expect not to flourish. This tipping point arguably marks the end of an adaptive low mood. At the outset of depression, low mood will often lead the person to take actions that improve their situation. Low mood can allow for the conservation and reallocation of energetic resources which can serve as an adaptive strategy for minimizing expected free energy in a threatening highly stressful, volatile, social environment (Barrett *et al.* 2016; Badcock *et al.* 2017; Clark *et al.* 2018). Rigid low mood however leads to what Badcock and colleagues have described as a ‘self-perpetuating cycle’, a positive feedback loop in which the persons samples the social world for sensory observations that confirm their expectation of suffering. One of the subjects (#75) Ratcliffe interviews tells him: ‘When I’m depressed life never seems worth living. I can never think about how my life is different when I’m not depressed. I think my life will never change and that I will always be depressed’ (Ratcliffe 2015, 69). The result of the rigidification of low mood is that the person doesn’t only experience a bad day every now and then, but they experience a loss of the possibility that the future could ever be good again.

First-person accounts of major depression often include descriptions of the person’s worldview shifting in important but difficult to express ways. A common experience that characterizes the phenomenology of major depression is a loss of depth, or a shrinking of the field of relevant affordances (De Haan *et al.* 2013). For the depressed person nothing seems interesting, no activity or event provokes a desire to engage. The ordinary scope for possible interactions with the world and others becomes greatly reduced. The world seems devoid of possibilities for improving their situation. Importantly, it is not only that the world lacks alluring things, it is that the possibility of being allured by affordances that contribute to the person’s well-being seems to have been altogether removed (Ratcliffe 2013, 586). The loss of this dynamic push-and-pull between the agent and the world that is ubiquitously felt in ordinary healthy experience produces a profound sense of estrangement—the depressed person and the world cease to fit together as they once had, the result is a feeling of strangeness, alienness or separateness. People will describe ‘being cut-off from an interpersonal world and stranded or incarcerated for all eternity in a different *kind* of world or reality’ (Ratcliffe 2015, 65).

This shrinking to nothing of significant possibilities can naturally be redescribed in terms of the affective structure of the field of relevant affordances as a whole (c.f. De Haan *et al.* 2013, 2015). We have shown above how inviting affordances stand out as relevant to an agent as a result of an increase in free energy the agent expects it can control through its actions. Precision we have been arguing, is estimated in part on the basis of error dynamics, and the expected deceleration or acceleration in free energy consequent on acting. The global downregulation of precision on affordances that happens through persistent low mood means that affordances that once solicited engagement will no longer invite as strongly, or at all. Notice however, that a pathological low mood in major depression signifies not only that one has lost confidence in particular affordances, but rather that there is a more *global* downregulation of expectations about any affordance succeeding at reducing error in the given environment. If the agent no longer has confidence in any of the affordances the environment offers them, this will have the consequence that they relate to a field of relevant affordances that is flat. This is arguably a difference in the kind of possibility the world can make available. The very possibility of anything standing out as significant is missing from their experience, and as a consequence, nothing calls them to action, except perhaps opportunities to end their life (Krueger and Colombetti 2018).

Badcock *et al.* (2017) propose that depressed mood can arise from the failure to attune to complex social niches, or more specifically, from the failure to share a reality with others. We suggest that a part of this social complexity might have to do with overly optimistic expectations for free-energy reduction, which the challenges of the person’s life circumstances conspire to frustrate. To offer another example, overly high expectations about success might be installed and sustained from family or culture. This would have the effect of setting precision weighting on a slope of error reduction that is much too high given the individual’s skills, abilities or environment. The result of not living up to those expectations would be negative surprise. In healthy and adaptive individuals, those errors would adjust precision expectations about the likelihood of future successes. However, depressed individuals, as we have already noted, learn to live in a world that offers only social distress. The failure to adjust to shifts in error dynamics can thus prove to be catastrophic for the person. When a person is bombarded by pathological negative surprise, or expects to always fail in their attempts at error reduction, they lose a sense of being able to improve in the ways that matter. The result is an experience of a world in which nothing matters—a world lacking in significance.

## Conclusion

The neurophenomenological account of mood we have outlined in this article is premised on a synergy of ideas drawn from the FEP, affective neuroscience and phenomenological philosophy. Looking at minds from this vantage point brings together the biological, psychological, phenomenological and the social. We began by sketching the phenomenological account of mood due to Ratcliffe. The phenomenology of mood can be described in terms of the kinds of significance a situation has for a person. Moods are existential feelings that structure and give style to a person’s lived experience. It is because of the mood a person is in that the situation they are in appears to have the significance it does—i.e. threatening, or foreboding, exciting or boring. We have set out to show how our ecological-enactive reading of the FEP provides tools for redescribing the phenomenology of mood in the terms of cognitive neuroscience. Mood we suggested can be described in terms of expectation of momentum in free-energy minimization. When in a positive mood, an individual will expect positive momentum. They will expect the environment to offer plentiful opportunities for new and exciting ways to live a fulfilling life. Thus positive mood biases expectations of how the agent will fare in its engagement with the world upwards. We suggest this is an example of how mood could structure the significance the individual experiences a situation as having.

Crucially, the individual’s positive mood will typically only continue as long as they continue to do well in keeping expected free energy to a minimum. If free energy begins to accelerate, this will typically lead to a change in mood for the worse. When in a negative mood, an individual will expect negative momentum—the acceleration in the increase of free energy. They will expect to fail at finding opportunities for improving grip which may lead them to employ tricks that help to lift their mood (e.g. meeting with a friend or playing music; Colombetti and Krueger 2015). Again this is an example of how mood might work to structure an individual’s sense of the significance of the situation they are in.

When mood becomes rigidified and ceases to keep up with the changing fortunes of an individual, the result can be a profound change in the possibilities the individual anticipates. In major depression for instance, a fixed low mood leads the person to experience a world emptied of its meaningfulness. Typically, precision is continually being tuned by our sensitivity to error dynamics, which we take to arise as part of our skilful engagement with the continually shifting field of affordances. The hopelessness experienced in major depression arises in part as a consequence of a breakdown in this process. In our ordinary healthy engagement with the world, we see the world as a place where we can progress, learn and grow. We are ‘built’ to grow in our skilled engagement with the world, and are happy and engaged only to the extent that we succeed in doing so.

## Acknowledgments

JK and ER are supported by the European Research Council in the form of ERC Starting Grant 679190 (EU Horizon 2020) for the project AFFORDS-HIGHER, the Netherlands Organisation for Scientific Research (NWO) in the form of a VIDI-grant awarded to ER, and by a project grant from the Amsterdam Brain and Cognition research group at the University of Amsterdam. MM carried out this work with the support of Horizon 2020 European Union ERC Advanced Grant XSPECT - DLV-692739.

*Conflict of interest statement*. None declared.
